# CMV retinitis in patients on mycophenolate immunosuppression: a report of two cases

**DOI:** 10.1186/s13223-023-00817-z

**Published:** 2023-08-19

**Authors:** Prem N. Patel, Ahmed M. Alkaliby, Mitul C. Mehta, Angeline L. Wang

**Affiliations:** 1 Department of Ophthalmology, UT Southwestern Medical Center, 5323 Harry Hines Blvd, Dallas, TX 75390-9057 USA; 2 Gavin Herbert Eye Institute, UC Irvine, Irvine, CA 92697 USA

**Keywords:** Cytomegalovirus, Mycophenolate, Retinitis

## Abstract

**Background:**

The rate of cytomegalovirus (CMV) retinitis is increasing, likely secondary to aggressive immunosuppressive regimens for a variety of diseases. Transplant and rheumatological literature show growing evidence suggesting a unique relationship between CMV infection and mycophenolate in particular. This study reports two cases of CMV retinitis infection in patients on mycophenolate immunosuppression.

**Case presentation:**

Case A was a 39-year-old African American woman with systemic lupus erythematosus (SLE) with stage IV lupus nephritis who presented for bilateral retinal detachments with areas of moth-eaten and thin retina concerning for prior viral retinitis. Case B was a 53-year-old man who presented with floaters in the right eye status-post heart transplant since 2008 on immunosuppressive therapy. Fundoscopic examination of the right eye showed frosted branch angiitis with intraretinal hemorrhage and inner retinal thickening and disorganization, consistent with CMV retinitis infection. Both patients were on mycophenolate immunosuppression with the recommendation to reduce or discontinue mycophenolate.

**Conclusion:**

Patients on mycophenolate immunosuppression may be more vulnerable to cytomegalovirus infection, including CMV retinitis. Ophthalmologists should be aware of this increased risk and consider reducing or discontinuing mycophenolate to promote viral clearance in these susceptible patients, in conjunction with the patient’s transplant or rheumatology teams.

## Background

Mycophenolate is a widely used immunosuppressive agent that inhibits inosine-5’-monophosphate, preferentially inhibiting B-cell and T-cell function [1]. The broad immunosuppressive function that makes mycophenolate efficacious for transplant patients and against autoimmune diseases may predispose to opportunistic infection. The most common infectious complication associated with mycophenolate use is cytomegalovirus (CMV) [2], which can lead to viral retinitis that presents with minimal intraocular inflammation, focal vasculitis, and granular retinal necrosis [3].

Several transplant and rheumatological studies have demonstrated an increased risk of cytomegalovirus infection specifically with mycophenolate immunosuppression compared to other agents [4–7]. This underlying vulnerability has yet to be thoroughly understood, but it is hypothesized that lower CMV IgM levels in these mycophenolate-treated patients may correlate with increased risk for infection [8]. We report two cases of CMV retinitis in patients on mycophenolate, with a discussion of the unique relationship between CMV and this immunosuppressive agent.

## Case presentation

### Case A

**Fig. 1:**
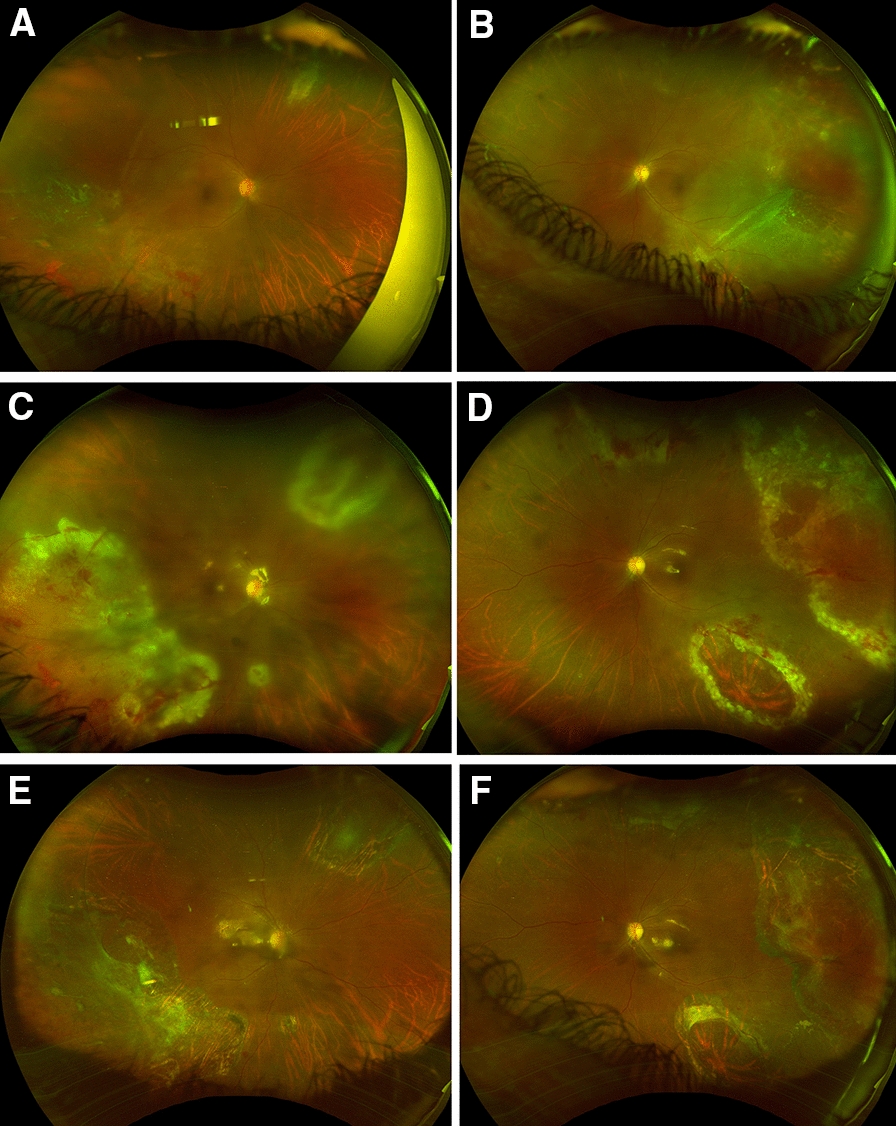
Fundus photography displaying Patient A’s clinical course, starting with characteristic necrotizing white lesion in the right **A** and left **B** eyes. Superior-temporal sub-retinal fluid and retinal detachment is present OU. **C**, **D** show reactivation of disease OU with new intraretinal hemorrhages and vasculitis following bilateral PPV with oil. The prior area of laser is surrounded with breaks and retinal atrophy. Repeat imaging at three months in the left **E** and right **F** eyes show resolution of scars at sites of previous lesions with minimal subretinal fluid

A 39-year-old African American woman with a history of systemic lupus erythematosus (SLE) was referred for bilateral retinal detachments. She presented with symptomatic floaters for 3 weeks and shadows for 1 week. Her best corrected visual acuity was 20/50 in the right eye and 20/30 in the left eye. Dilated fundus exam revealed bilateral inferotemporal detachments involving the macula with large moth-eaten and thin retina with multiple holes (Fig. 1A, B). The patient underwent left eye retinal detachment repair with pars plana vitrectomy and silicone oil placement on the day of presentation, with right eye retinal detachment repair performed 1 week later.

Four months prior to presentation, the patient developed lupus nephritis, prompting an increase of her oral prednisone dose from 10 mg daily to 60 mg daily, as well as the addition of high-dose mycophenolate mofetil at 3 g daily.

Given that the exam findings were concerning for previous viral retinitis, viral polymerase chain reaction (PCR) of the vitreous fluid was sent for the left eye on the day of the first surgery. The vitreous fluid was positive for CMV. The patient was also found to have active CMV viremia with a serum CMV PCR of 18,300 IU/mL (normal < 137 IU/mL). The patient was started on induction therapy with IV ganciclovir at 150 mg twice daily for 3 weeks, then switched to oral valganciclovir 900 mg daily for CMV maintenance therapy.

The patient was noted to have recurrence of CMV retinitis in both eyes the day after her second surgery with anterior chamber inflammation, retinal hemorrhages and vasculitis in both eyes mainly in the areas of previous retinal thinning (Fig. 1C, D). The retinitis slowly improved on systemic valganciclovir 900 mg daily. In addition, per recommendations from Infectious Disease, the patient’s mycophenolate mofetil dose was reduced to standard dose of 2 g per day and oral prednisone reduced to 15 mg daily.

The patient remains fully attached in both eyes 16 months following retinal detachment repair. Her CMV retinitis has not recurred, and she remains on prophylactic valganciclovir 900 mg daily. Fundus photography showed resolving scars at the sites of previous lesions (Fig. 1E, F).

## Case B

**Fig. 2:**
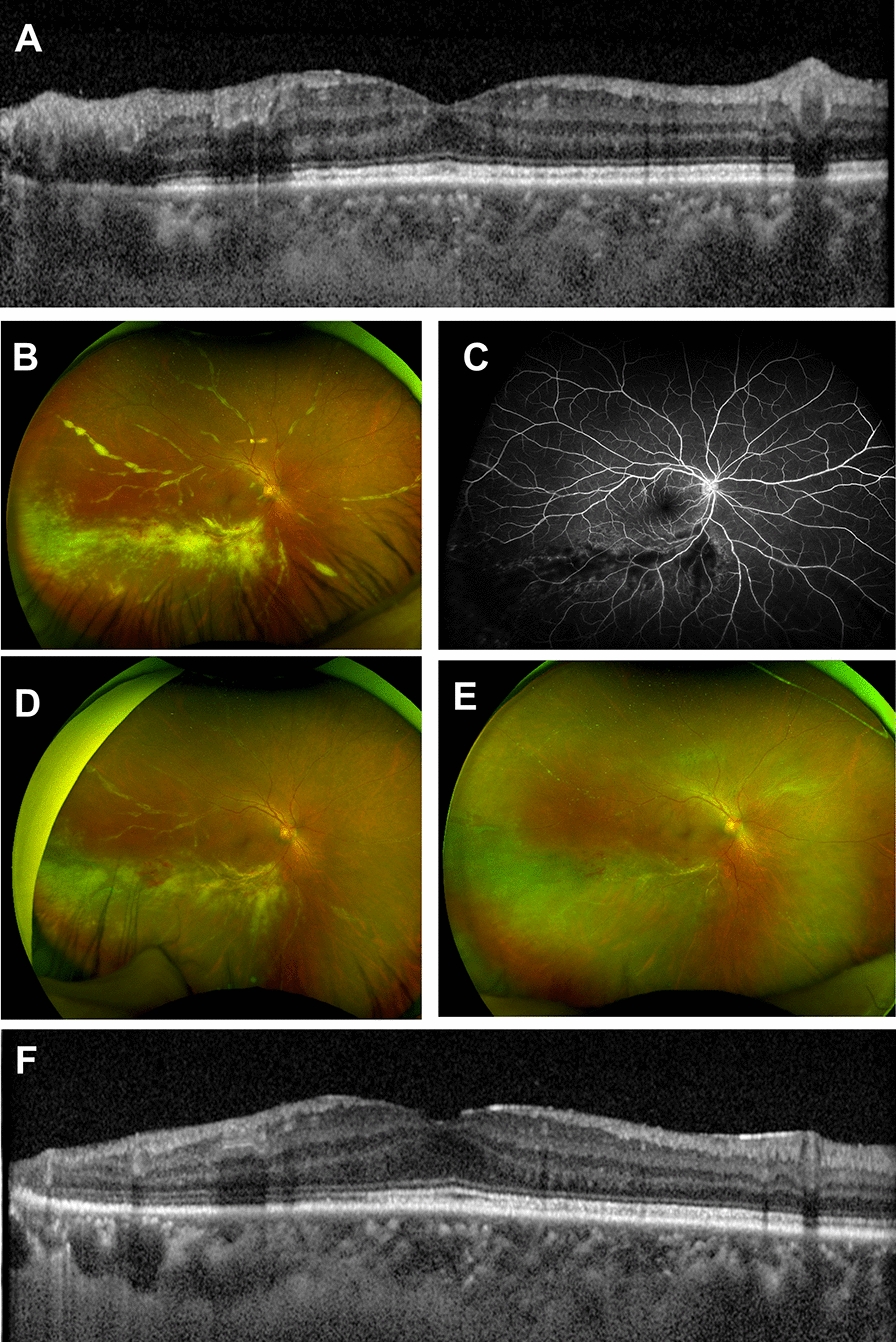
**A** Optical coherence tomography of Patient B showing inner retinal thickening disorganization of the inferior macula. **B** Fundus photography OD at presentation displaying classic frosted branch angiitis, with hemorrhage and white lesions surrounding the retinal vessels. **C** Fluorescein angiography shows leaking vessels in involved areas of retina. **D** Fundus imaging after partial treatment (2 weeks after presentation), shows improvement following second Foscarnet injection. **E** 1 year fundus photography showing improved vasculitis, resolved intraretinal hemorrhage, and resolved retinal whitening. **F** 1 year OCT shows stable atrophy inferotemporally

A 53-year-old Caucasian man with a history of congestive heart failure status post heart transplant about 10 years prior presented with 2 days of blurred vision and floaters in the right eye. His best-corrected visual acuity was 20/30 in the right eye and 20/20 in the left eye. Dilated funduscopic exam of the right eye demonstrated frosted branch angiitis of the inferior arcade with hemorrhages and whitening of the inferior retina (Fig. 2B). Spectral-domain optical coherence tomography (SD-OCT) (Fig. 2A) demonstrated inner retinal thickening and disorganization in the areas of retinitis. Fundus exam of the left eye was unremarkable. At the time, patient was taking 360 mg of mycophenolate sodium daily, 5 mg prednisone daily, and 1 mg tacrolimus twice daily, 25 mg atenolol twice daily, 40 mg atorvastatin daily, and 300 mg allopurinol daily.

Exam findings were consistent with CMV retinitis in the clinical setting of systemic immunosuppression for heart transplant. The patient’s transplant surgeon was contacted, who strongly recommended switching immunosuppression from mycophenolate sodium to leflunomide 20 mg/day due to concerns about CMV clearance on mycophenolate.

Foscarnet (2.4 mg in 0.1 ml per injection) was injected twice per week for 2 weeks, then weekly until quiescent, and valganciclovir 900 mg oral twice daily was also initiated. The patient’s exam findings of frosted branch angiitis and retinal hemorrhage improved after each injection and resolved completely after the 7th injection (Fig. 2D). A total of 8 injections were completed. The patients’ valganciclovir was also decreased to 450 mg daily per infectious disease recommendation due to decreased creatine clearance.

The right eye remained quiet with no recurrence of retinitis for follow up period of 1 year. Fundus examination revealed improved vasculitis, resolved IRH, and resolved retinal whitening (Fig. 2E). Optical coherence tomography (OCT) showed stable atrophy inferotemporally (Fig. 2F).

## Discussion and conclusions

Cytomegalovirus (CMV) is a *Herpesviridae* virus that classically infects hosts with compromised immunity, especially in patients with HIV, hematological malignancy, and organ transplantation [4]. However, CMV infection also occurs in the setting of aggressive immunosuppression. Among the immunosuppressive agents, mycophenolate is recognized by transplant literature as a significant risk factor for CMV disease [5]. One clinical trial found that 3 g/day of mycophenolate mofetil with conventional cyclosporine and steroids had a greater incidence of CMV (35.7%) compared to placebo or a standard dose of 2 g/day (< 8%).^6^ Another transplant study found that mycophenolate use was associated with a greater frequency of organ involvement, suggesting increased morbidity of CMV infection [7]. Mycophenolate is believed to induce specific changes in the primary immune response by suppressing CMV IgM production [8], and IgM response is inversely correlated with CMV disease severity. These observations altogether support a special relationship between mycophenolate use and the incidence and tissue-invasiveness of CMV disease.

CMV may invade hematogenously to the eye and cause CMV retinitis, which presents as a progressive necrotizing retinitis with a well-characterized clinical course. Typically, CMV retinitis occurs in AIDS patients, but cases are being increasingly reported in patients on two or more immunosuppressive agents at a time. Regarding mycophenolate, patients with CMV retinitis have been kept on standard dosing of 2 g/day mycophenolate mofetil [9], and in other cases, clinicians have decided to discontinue lower doses of 1 or 1.5 g/day mycophenolate mofetil [10, 11]. In these cases, first line therapy of antiretroviral therapy was used, and retinitis was successfully resolved without complication.

Pertaining to the present report, Patient A was on a high-dose 3 g/day mycophenolate mofetil regimen due to the development of lupus nephritis 5 months prior. In her clinical course, mycophenolate was lowered to 2 g/day. In contrast, Patient B was switched off mycophenolate to leflunomide. Both patients presented with typical fundoscopic findings, absence of systemic CMV disease, and a positive CMV PCR, which all supported our diagnosis of CMV retinitis in the setting of immunosuppression.

The specific role that mycophenolate played in the development of CMV retinitis in our report, and in those previously mentioned, remains unclear for several reasons. First, these patients are typically on a variety of immunosuppressive agents and monotherapy is uncommon, making generalization difficult. Second, it is possible that two or more agents produce a synergistic effect towards CMV retinitis susceptibility. Such may be the case with Patient A, who was also on high-dose prednisone for her lupus nephritis flare. A similar patient was reported to develop CMV retinitis while on mycophenolate mofetil, tacrolimus, and low-dose corticosteroids [12]. Our report is not without limitations, such as the absence of HIV serology and CD4 count in Patient A or B. While these tests are preferable to rule out alternative etiologies of immunocompromise, they were not performed due to low clinical suspicion from the Infectious Disease service. However, it is reassuring to note that neither Patient A or B has developed signs or symptoms of HIV infection since the present report, to the best of our knowledge.

To sum, we report two cases of CMV retinitis in patients taking mycophenolate as immunosuppression in the setting of SLE and post-organ transplant. The transplant and rheumatology literature outline a unique susceptibility to CMV disease in mycophenolate-treated patients. Thus, when mycophenolate is used, ophthalmologists should be more aware of CMV retinitis reactivation and disease.

## Data Availability

Not applicable.

## Abbreviations

CMV: Cytomegalovirus
HIV: Human immunodeficiency virus
SLE: Systemic lupus erythematosus
PCR: Polymerase Chain Reaction
OCT: Optical coherence tomography
SD-OCT: Spectral-domain optical coherence tomography
